# Descriptive Analysis of Adverse Drug Reactions Reports of the Most Consumed Antibiotics in Portugal, Prescribed for Upper Airway Infections

**DOI:** 10.3390/antibiotics11040477

**Published:** 2022-04-02

**Authors:** Joana Ferreira, Ana Isabel Placido, Vera Afreixo, Inês Ribeiro-Vaz, Fátima Roque, Maria Teresa Herdeiro

**Affiliations:** 1 NOVA Medical School, Faculty of Medical Sciences, New University of Lisbon (NMS|FCM UNL), 1169-056 Lisbon, Portugal; joana.ferreira.555@gmail.com; 2 Research Unit for Inland Development, Polytechnic of Guarda (UDI-IPG), 6300-559 Guarda, Portugal; anaplacido@ipg.pt; 3 Center for Research & Development in Mathematics and Application (CIDMA), University of Aveiro, 3810-193 Aveiro, Portugal; vera@ua.pt; 4 Porto Pharmacovigilance Centre, Faculty of Medicine, University of Porto, 4200-450 Porto, Portugal; inesribeirovaz@gmail.com; 5 Center for Health Technology and Services Research (CINTESIS), Faculty of Medicine, University of Porto, 4200-450 Porto, Portugal; 6 MEDCIDS—Department of Community Medicine, Health Information and Decision, Faculty of Medicine, University of Porto, 4200-450 Porto, Portugal; 7 Health Sciences Research Centre, University of Beira Interior (CICS-UBI), 6200-506 Covilhã, Portugal; 8 Institute of Biomedicine, Department of Medical Sciences, University of Aveiro, 3810-193 Aveiro, Portugal; teresaherdeiro@ua.pt

**Keywords:** adverse drug reactions, antibiotics, upper airway infections, EudraVigilance database

## Abstract

Adverse drug reactions (ADR) significantly impact mortality and morbidity and lead to high healthcare costs. Reporting ADR to regulatory authorities allows for monitoring the safety and efficacy profile of medicines on the market and for assessing the benefit–risk ratio. This retrospective study aims to characterize the ADR profile of the most consumed antibiotics in Portugal that are prescribed for upper airway infections and submitted to the EudraVigilance database. The variables were analyzed in an exploratory perspective, through absolute and relative frequencies, with emphasis on serious ADR. A total of 59,022 reports were analyzed of which 64.4% were classified as suspected serious ADR. According to serious ADR, the female sex (52.2%) and 18–64 age group (47.5%) prevail. Health professionals reported 87.8% of suspected serious ADR and European Economic Area (EEA) countries represented 50.8% of the reports. “Skin and subcutaneous tissue connections” (15.9%), “general disorders and administrations site conditions” (12%), and “gastrointestinal disorders” (9.8%) are the prevalent system organ classes. In 4.5% of the reports, patients had a fatal outcome. A periodic evaluation of the safety of the antibiotic should be performed to facilitate the development of guidelines and policies to reduce the frequency of serious ADR.

## 1. Introduction

Although a broad range of antimicrobial stewardship programs was developed [1,2] and implemented, antibiotics still rank as one of the most consumed types of medicine worldwide (ranging from 4.4 to 64.4 defined daily doses (DDD) per 1000 inhabitants [3]. In Europe, between 2019 and 2020 there was an overall decrease in the population-weighted mean total (community and hospital sectors combined) consumption of antibacterials for systemic use from 19.9 DDD per 1000 inhabitants per day to 16.4 DDD per 1000 inhabitants per day in 2020 [3].

In Portugal, in 2019, the median antibiotic use was 19.3 DDDs per 1000 inhabitants per day with a slight upward trend in comparison with the two previous years (2017: 18.3 DDD per 1000 inhabitants; 2018: 19.1 DDD per 1000 inhabitants) [3]. This trend was reversed in 2020 with overall antibiotic use of 15.2 DDD per 1000 inhabitants [3]. The decrease in total antibiotic consumption in Portugal in 2020 (13.7 DDD per 1000 inhabitants) is due to the decrease in antibiotic consumption at the community level. At the hospital level, an increase of 0.8% was observed in the yearly growth rate [3].

Antimicrobial resistance is strongly associated with the overuse of antibiotics and is one of the greatest threats to public health, not only in developing countries but also worldwide [4,5,6,7,8]. Aside from antimicrobial resistance, the inappropriate use of antibiotics is also a major issue as antibiotics can be linked to a large number of adverse drug reactions (ADR), including allergic reactions, end-organ toxicity, further infections by an antibiotic-resistant organism, or even death [3,9,10,11].

In this context, pharmacovigilance systems are essential to assess and monitor the safety of human medicines and to provide consistent data for an effective evaluation of the risks and benefits of the use of a drug [12].

Respiratory tract infections are among the most frequent causes of hospitalization and death among adults [13,14,15]. Whereas 40–50% of respiratory infections are viral, antibiotic therapy has often been used to treat this condition [16,17].

While it has been suggested that antibiotics only slightly modify the evolution of respiratory tract infections [7,18,19,20], antibiotics account for nearly 60% of all prescriptions within a primary care setting [10,11,21,22].

Therefore, we sought to analyze the ADR profile of the most used antibiotics in Portugal, appropriated for the treatment of upper airway infections through the analysis of the European system for managing and analyzing information on suspected adverse reactions to medicines EV.

## 2. Results

### 2.1. Analysis of the complete Data set

Between 2017–2019, a total of 59,022 suspected ADR reports were associated with the most used antibiotics in both ambulatory (amoxicillin+ clavulanic acid; azithromycin, amoxicillin, ciprofloxacin, and clarithromycin) and hospital (amoxicillin+ clavulanic acid, ciprofloxacin, cefazolin, azithromycin, and levofloxacin) settings. Moreover, the number of reports has risen over the years (Table 1). Among the total number of suspected ADR reports, 55.0% occurred in women, 40% in men, and the remainder did not specify sex.

It was also observed that more than 50.0% of the suspected ADR reports were related to the combination of amoxicillin and clavulanic acid (26.3%), and amoxicillin (20.9%). Cefazolin has the lowest number of suspected ADR reports (2.7%) (Table 1). Regarding the age group, it was observed that 48% of the retrieved suspected ADR reports belonged to the age group 18–64. Finally, the primary sources of the majority of the suspected ADR reports were health professionals (82% of all reports).

### 2.2. Characterization of Suspected Serious ADR

The suspected serious ADR reports represent 64.4% (37,7982 reports) of all suspected ADR reports; 50.8% of them are from European Economic Area (EEA) countries ranging from 3.5% for cefazoline to 28.9% for amoxicillin+ clavulanic acid. Non-EEA countries accounted for 49.2% of serious suspected ADR reports (range from 2.8 for cefazoline to 31.7 for levofloxacin). Serious suspected ADR reports correspond to a total of 165,408 suspected ADR (72.6% of all ADR reports). The mean range of serious suspected ADR described for each report ranges from 2.6 amoxicillin to 4.4 for ciprofloxacin.

The subjects of the 19,824 (52.2%) suspected serious ADR reports were female, 15,695 (41.3%) males, and 6.5% of the cases did not specify sex. It was also observed that for children under 12 years old, (i.e., 0–11 years) reports referring to male subjects are predominant; this trend changes in the remaining age groups (Table 1).

The highest number of suspected serious ADR reported for all the analyzed active substances was observed from adult subjects (18–64 years). Among the reported suspected serious ADR, cefazoline (3.5%) was the least reported, and amoxicillin+ clavulanic acid was the most reported (25.4%). At younger ages (0–18 years), a total of 3182 (52.3%) suspected serious ADR reports were reported and were related to amoxicillin+ clavulanic acid, azithromycin, and amoxicillin.

Otherwise, in older adults (65+) only 31.2% of the suspected ADR reports were classified as serious. Levofloxacin and the combined substances of amoxicillin+ clavulanic were the most prevalent active substances reported, corresponding to a total of 28.0% and 23.3% of all suspected serious ADR.

### 2.3. System Organ Class Level

**Figure 1:**
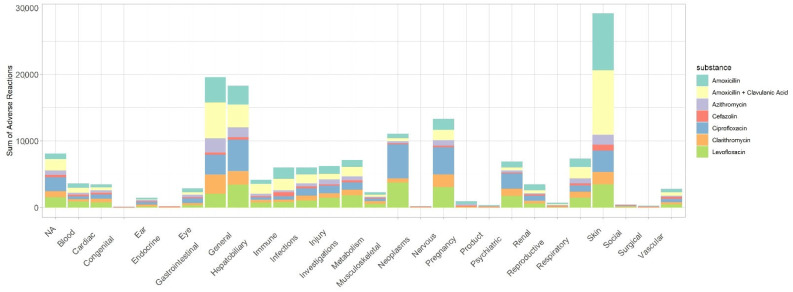
Suspected serious ADR distributed by the different system organ class for each active substance.

According to Figure 1, the SOC “Skin and subcutaneous tissue disorders”, “general disorders and administration site conditions”, and “gastrointestinal disorders” present the highest values of suspected serious ADR.

Moreover, in the age group from 18 to 64 years old, these SOC represented approximately 60% of suspected serious ADR (Table 2). Finally, it was also observed that “skin and subcutaneous tissue disorders”, “general disorders and administration site conditions”, and “gastrointestinal disorders” present represented approximately 39% and 38% of suspected serious ADR in females and males, respectively.

A detailed analysis of the three most frequent SOCs (“Skin and subcutaneous tissue disorders”, “general disorders and administration site conditions”, and “gastrointestinal disorders”), revealed that the PT diarrhea and vomiting (SOC gastrointestinal disorders) and urticaria and erythema (Skin and subcutaneous tissue disorders) were most frequently observed to the combination amoxicillin+ clavulanic acid suspected ADR reports. It was also observed that “rash” was the most frequently PT related to amoxicillin among suspected ADR reports. In clarithromycin suspected ADR reports it was observed that “drug interactions” and “nausea” were the most frequent PT (Table 3).

The most prevalent outcome observed for serious ADR were “other medically important condition” (48.4%) and “hospitalization” (initial or prolonged) (36.2%). The serious ADR criterion “life-threatening” was observed in 7.4% and “death” in 4.5% of the reports. Clinical conditions such as “disability” and “congenital anomaly” were observed in 3.4% and 0.1% of the reports, respectively.

“Death” and “hospitalization” (initial or prolonged) were more frequent in males than in females, and “other medically important condition” was more prevalent in females (Table 4). “Hospitalization” (initial or prolonged) occurs predominantly in the older (65–85 years) and very older adults (+85 years). A total of 45% of the deaths were from adult subjects (18–64 years) and 32% in older adults (65–85 years) (Table 4).

## 3. Discussion

To the best of our knowledge, this is the first study analyzing the EV database, the seriousness of suspected ADR in the most-used antibiotics used for treatment of upper airway infections in the Portuguese population. It was observed that the majority of the suspected ADR reports classified as serious were reported by health professionals and were associated with adult female subjects. “Skin and subcutaneous tissue disorders”, “general disorders and administration site conditions”, and “gastrointestinal disorders” were the SOC of most of the suspected serious ADR. One-half of the suspected serious ADR had as seriousness criterion the “other medically important condition”.

In 2019 it was observed that in Portugal the use of antibiotics for systemic use was lower than the EU/EEA mean total consumption [3]. However, beta-lactam/penicillin and macrolides, lincosamides, and streptogramins consumption was higher than the EU/EEA average [3]. According to data from health market research system (hmR 2020) it was observed that amoxicillin and amoxicillin+ clavulanic acid, azithromycin, and clarithromycin were the most used antibiotics in ambulatory care in Portugal. 

Between 2017–2019, an increase of 41% occurred in the number of suspected ADR reports for the active substances included in this study. This increasing trend was also observed by others [23,24,25,26].

The EV database does not allow for categorizing reports by health professionals, but according to the literature, despite reporting few cases [27], physicians were the most active players as the primary source of ADR reports [23,25,28]. A recent review suggests that training health professionals is essential to improving the number of reported ADR [29].

The rate of ADR reported by non-health professionals (patients and other sources) is higher than observed in other studies [23,25,28,29]. According to Dubrall et al., the increase over the years in the number of ADR reported by non-health professionals can be related to an increase in ADR reported by patients [25].

Health professionals tend to report ADR based on clinical data, and non-health professionals sustained their ADR report in the outcomes that impaired their daily routine [25].

In this study, it was observed that suspected ADR reports associated with females were prevalent, and identical patterns of distribution of ADR reports by sex have been reported by others [12,25,30,31,32]. Iftikhar et al. (2018) [33], observed a predominance of ADR reports in males; however, its study was conducted in hospitalized patients.

In this study, it was observed that the majority of the reported suspected ADR were classified as serious and this fact could be probably related to increasingly strict legal reporting requirements in the industry [34]. The analysis of the geographic localization of ADR revealed that in EEA countries there was an equative distribution between the number of serious/non-serious reports ADR. In non-EEA countries, 98.4% of the reported cases were referred to seriously as ADR. This data can be justified by the obligation of the non-EEA countries to report all serious ADR and EEA countries must report all ADR independently of seriousness [33,34,35].

Following previous studies [36], it was observed that in children below 12 years the majority of ADR reports belong to male subjects. This predominance of ADR in male subjects can be associated with the different patterns of exposure to drugs as some childhood diseases and infections occur more frequently among young boys than girls or have an influence on hormonal factors [24,30,37,38,39].

In this study, it was observed that 30% of the suspected ADR were from older adults (≥65 years). which can be due to the presence of pharmacokinetics and pharmacodynamic alterations resulting from aging [33,40].

The high number of serious ADR observed in females can be the result of the pharmacokinetics differences associated with sex or the high number of medicines used by females [33,41,42].

The SOC with the highest prevalence of suspected ADR were “Skin”, “General”, and “Gastrointestinal”; similar results were observed by others [23,24,26,33,40,42]. These were more prevalent in females and older adults. The slight differences of SOCs related to age were following the literature due to the different populations (children, adults, or older adults), size of the study population and active substance analyzed [23,24,25,26,42].

Previously, studies had observed that reactions such as “diarrhea”, “rash”, “pruritus”, and “urticaria” were associated with antibiotics of systemic use [27]. Moreover, reactions such as “nausea”, “dyspnea”, and “pyrexia” were described as frequent in the adult population, and “pyrexia”, vomiting, and convulsion were more related to the pediatric population [28].

As previously observed, the combination of amoxicillin and clavulanic acid is associated with an increased risk of gastrointestinal ADR, causing symptoms such as “diarrhea” [43]. “Hospitalization” (initial or prolonged) was associated with more than 35% of the serious suspected ADR, a number slightly higher than observed by others [24,42]. A recent study performed in hospitalized patients revealed that ADR associated with antibiotics use were the most commonly involved medication class associated with hospitalization [43].

Regarding the outcome of “death”, this outcome was observed in 4.5% of the serious suspected ADR, a number higher than observed by others [41]. We also observed that “death” and “hospitalization” (initial or prolonged) are more frequent in males. Otherwise, a “medically important event or reaction” was more frequently observed in females.

As with other retrospective ADR studies, this study has some limitations related to the incomplete information of the suspected ADR reports. Moreover, the EV database does not allow access to individual information, so the data were conditioned to group analysis [44]. Furthermore, in other similar studies that use spontaneous reporting data, the results are hampered by underreporting, overreporting, and reporting bias [45]. Another limitation is related to the fact that the data cannot be used to determine the possibility of having an ADR and that cases are reported based on a suspicion of a causal relationship between drug intake and ADR, which does not necessarily mean that causality has been established.

## 4. Materials and Methods

### 4.1. Data Source and Definition Dataset

A retrospective study was conducted to evaluate suspected ADR of the most used antibiotics in Portugal appropriated for treatment of upper airway infections in both ambulatory care and hospitals between 1 January 2017 and 31 December 2019, reported in the centralized system, authorized in the EEA for managing and analyzing information on suspected adverse reactions to EV [46]. The European Medicines Agency (EMA) operates the system on behalf of the European Union (EU) medicines regulatory network.

In the EV database suspected ADR are coded with MedDRA terminology, the Medical Dictionary for Regulatory Activities (MedDRA) coding system, which is organized in 27 System Organ Classes (SOC) refers to a group of MedDRA terms belonging to a SOC [47]. Suspected ADR can be analyzed according to the SOC level, i.e., the organ system in which the suspected ADR occurs, or on a more detailed level such as the preferred term (PT), which coded information relates to the reported symptoms.

The data regarding the most used antibiotics in ambulatory settings was obtained through the analysis of the percentage of consumed active substances from the information system of the health market research (hmR 2020). All the active substances included in this study were classified following the anatomical Therapeutic Chemical (ATC) classification of WHO and belonging to the J01- antibacterials for systemic use group [48].

The ranking of the most used antibiotics in the hospital was estimated after the analysis of the report published by the government agency accountable to the Portuguese Health Ministry [49].

Only active substances with a clinical indication for upper airway infections described in the summary of product characteristics were considered in this study [50]. EV database was accessed on May 2020 and information regarding suspected ADR of the selected substances were extracted. Extracted data included: active substance, reaction groups, patients’ sex, age group, geographic origin, gateway year, reporter group (non-/healthcare professional), and seriousness. A suspected ADR is classifieds as serious if “it corresponds to a medical occurrence that results in death, is life-threatening, requires inpatient hospitalization, results in another medically important condition, or prolongation of existing hospitalization results in persistent or significant disability or incapacity or is a congenital anomaly/birth defect” [51].

### 4.2. Statistical Analysis

Data were collected from the EV database and statistically analyzed with the package epiR of the program R (version R 3.6.2). Categorical variables were presented in frequencies. To general data, the total number of reported ADR reports was the denominator of the descriptive analysis. To a detailed analysis of serious ADR, the denominator was the total number of serious ADR reported. The results were expressed with 95% confidence intervals (CI95).

To avoid multiple entries of the same suspected ADR report, each suspected ADR was assigned to seriousness criteria: (a) Death; (b) Life-Threatening; (c) Hospitalization (initial or prolonged); (d) Disabling/Incapacitating; (e) Congenital Anomaly; (f) Other Medically Important Condition [52].

## Figures and Tables

**Table 1:** Annual characterization of EudraVigilance reports of amoxicillin, amoxicillin + clavulanic acid, azithromycin, cefazolin, clarithromycin, and levofloxacin.

		Amoxicillin		Amoxicillin + Clavulanic Acid		Azithromycin		Cefazolin		Ciprofloxacin		Clarithromycin		Levofloxacin		Total	
		Total (%)	Serious (%)	Total (%)	Serious	Total (%)	Serious (%)	Total (%)	Serious (%)	Total (%)	Serious (%)	Total (%)	Serious (%)	Total (%)	Serious (%)	Total (%)	Serious (%)
Year	2017	3180 (19)	2329 (18)	4406 (26)	3069 (24)	1120 (7)	853 (7)	580 (3)	458 (4)	2777	2153 (17)	1601 (9)	1188 (9)	3239 (19)	2674 (21)	**16,903** **(29)**	**12,724** (33)
	2018	4041 (22)	1981 (19)	5242 (29)	3069 (29)	1247 (7)	641 (6)	466 (3)	335 (3)	2963	1709 (16)	1666 (9)	855 (8)	2740 (15)	1891 (18)	**18,365** **(31)**	**10,481** (28)
	2019	5115 (22)	2772 (19)	5867 (25)	3418 (23)	1711 (7)	1011 (7)	544 (2)	414 (3)	3606	2181 (15)	1958 (8)	1033 (7)	4953 (21)	3948 (27)	**23,754** **(40)**	**14,777** (39)
	Total	**12,336**	**7082**	**15,515**	**9556**	**4078**	**2505**	**1590**	**1207**	**9346**	**6043**	**5225**	**3076**	**10,932**	**8513**		
Sex	Male	4603 (19)	2639 (17)	6228 (26)	3891 (25)	1492 (6)	994 (6)	704 (3)	525 (3)	4083 (17)	2640 (17)	1743 (7)	1087 (7)	5036 (21)	3919 (25)	**23,889** **(40)**	**15,695** (41)
	Female	7218 (22)	4013 (20)	8322 (29)	4856 (24)	2396 (7)	1355 (7)	848 (3)	649 (3)	4933 (15)	3147 (16)	3230 (10)	1790 (9)	5257 (16)	4014 (20)	**32,204** **(55)**	**19,824** (52)
	Not specified	515 (18)	430 (17)	965 (25)	809 (33)	190 (6)	156 (6)	38 (1)	33 (1)	330 (11)	256 (10)	252 (9)	199 (8)	639 (22)	580 (24)	**2929** **(5)**	**2463** (6)
Age Group	0–1 month	22 (26)	18 (28)	26 (31)	18 (28)	19 (23)	16 (25)	5 (6)	4 (6)	5 (6)	3 (5)	4 (5)	2	3 (4)	3 (5)	**84** **(0)**	**64** (0)
	2 months–2 years	848 (44)	324 (38)	661 (35)	289 (34)	215 (11)	149 (17)	15 (1)	9 (1)	38 (2)	23 (3)	116 (6)	47	19 (1)	16 (2)	**1912** **(3)**	**857** (2)
	3–11 years	952 (35)	413 (29)	921 (34)	463 (33)	379 (14)	245 (17)	33 (1)	19 (1)	106 (4)	82 (6)	279 (10)	153	46 (2)	43 (3)	**2716** **(5)**	**1418** (4)
	12–17 years	390 (28)	216 (26)	421 (31)	246 (29)	139 (10)	80 (9)	48 (4)	35 (4)	116 (8)	84 (10)	120 (9)	74	136 (10)	108 (13)	**1370** **(2)**	**843** (2)
	18–64 years	5627 (20)	3307 (18)	7481 (27)	4584 (25)	1879 (7)	1074 (6)	826 (3)	626 (3)	4760 (17)	3059 (17)	2507 (9)	1413	5055 (3592)	3966 (22)	**28,135** **(48)**	**18,029** (47)
	65–85 years	2372 (17)	1429 (15)	3303 (23)	2119 (22)	687 (5)	437 (5)	499 (3)	385 (4)	2645 (18)	1763 (18)	1212 (8)	789	3592 (25)	2713 (28)	**14,310** **(24)**	**9635** (25)
	More than 85	499 (16)	326 (15)	967 (31)	642 (29)	108 (3)	83 (4)	68 (2)	45 (2)	518 (16)	370 (17)	200 (6)	139	798 (25)	606 (27)	**31,58** **(5)**	**2211** (6)
	Not specified	1626 (22)	1049 (21)	1735 (24)	1195 (24)	652 (9)	421 (9)	96 (1)	84 (2)	1158 (16)	659 (13)	787	459	1283 (17)	1058 (21)	**7337** **(12)**	**4925** (13)
Reporter group	Health professional	10,152 (21)	6444 (19)	13,422 (28)	8765 (26)	2929 (6)	2026 (6)	1563 (3)	1188 (4)	6648 (14)	4577 (14)	3857 (8)	2581 (8)	9609 (20)	606 (27)	**48180** **(68)**	**33,350** (88)
	Other	2184 (20)	20	2093 (19)	19	1149 (11)	11	27 (0)	0	2698 (25)	25	1368 (13)	13	1323 (12)	1058 (21)	**10,842** **(32)**	**4638** (12)
Geographic origin	EEA	9552 (24)	4368 (23)	11495 (29)	5571 (29)	2390 (6)	852 (4)	1055 (3)	678 (4)	6773 (17)	3509 (18)	3830 (10)	1726 (9)	4935 (12)	2581 (13)	**40,030**	**19,285** (51)
	Non-EEA	2784 (15)	2714 (15)	4020 (21)	3985 (21)	1688 (9)	1653 (9)	535 (3)	529 (3)	2573 (14)	2534 (14)	1395 (7)	1350 (7)	5997 (32)	5932 (32)	**18,992** **(32)**	**18,697** (49)

EEA—European Economic Area.

**Table 2:** Distribution of the SOC skin and subcutaneous tissue disorders, general disorders and administration site conditions, and gastrointestinal disorders by age and sex.

	Age Group							Sex	
MedDRA SOCs	0–1 Month (%)	2 Months–2 Years (%)	3–11 Years (%)	12–17 Years (%)	18–64 Years (%)	65–85 Years (%)	More than 85 Years (%)	Male (%)	Female (%)
Gastrointestinal disorders	7 (0.1)	242 (2.4)	452 (4.5)	280 (2.8)	5989 (59.9)	2614 (26.1)	420 (4.2)	4057 (8.7)	6668 (10.7)
General disorders and administration site conditions	16 (0.1)	233 (1.9)	527 (4.3)	294 (2.4)	7454 (60.4)	3314 (26.9)	495 (4.0)	5674 (12.1)	7392 (11.9)
Skin and subcutaneous tissue disorders	13 (0.1)	611 (3.7)	1059 (6.4)	516 (3.1)	9290 (56.6)	4034 (24.6)	902 (5.5)	7089 (15.2)	9966 (16.0)

MeDRA SOCs- Medical Dictionary for Regulatory Activities System Organ and Classes.

**Table 3:** Most frequent Preferred Term according to the most prevalent system organ and class or Amoxicillin, Amoxicillin + clavulanic acid, azithromycin, cefazolin, ciprofloxacin, clarithromycin, and levofloxacin.

		General Disorders and Administration Site Conditions			General Disorders and Administration Site Conditions			Skin and Subcutaneous Tissue Disorders			
	Preferred Term	Diarrhea	Nausea	Vomiting	Pyrexia	Drug Ineffective	Drug Interaction	Pruritus	Rash	Urticaria	Erythema
Active Substances											
Amoxicillin		267	206	222	234	105	81	498	1060	538	256
Amoxicillin + clavulanic acid		619	309	466	353	295	80	626	1289	762	430
azithromycin		179	126	129	112	206	90	120	168	86	47
cefazolin		10	13	14	34	30	13	41	81	49	61
ciprofloxacin		305	319	173	215	299	242	218	344	143	149
clarithromycin		207	200	165	126	140	298	99	164	95	80
levofloxacin		203	265	261	293	220	154	390	551	202	178

**Table 4:** Distribution of suspected ADR reports related to seriousness by age, sex, and active substance.

		Death (%)	Life-Threatening (%)	Hospitalization (Initial or Prolonged) (%)	Persistent or Significant Disability or Incapacity (%)	Congenital Anomaly (%)	Medically Important Event or Reaction (%)
Amoxicillin		126 (1.8)	490 (6.9)	2495 (35.2)	64 (0.9)	13 (0.2)	3894 (55.0)
Amoxicillin + Clavulanic acid		358 (3.7)	711 (7.4)	3464 (36.2)	101 (1.1)	8 (0.1)	4914 (51.4)
Azithromycin		128 (5.1)	134 (5.3)	869 (34.7)	56 (2.2)	7 (0.3)	1311 (52.3)
Cefazoline		62 (5.1)	332 (27.5)	419 (34.7)	3 (0.2)	1 (0.1)	390 (32.3)
Ciprofloxacin		287 (4.7)	360 (6.0)	2152 (35.6)	487 (8.1)	5 (0.1)	2752 (45.5)
Clarithromycin		127 (4.1)	168 (5.5)	1091 (35.5)	83 (2.7)	3 (0.1)	1604 (52.1)
Levofloxacin		632 (7.4)	621 (7.3)	3249 (38.2)	491 (5.8)	3 (0.0)	3517 (41.3)
Age groups	0–1 month	6 (9)	8 (12)	21 (33)	2 (3)	1 (2)	26 (4)
	2 months–2 years	23 (3)	30 (4)	305 (36)	8 (12)	9 (1)	487 (57)
	3–11 years	33 (2)	77 (5)	499 (35)	15 (1)	6 (0)	788 (56)
	12–17 years	17 (2)	72 (9)	330 (39)	14 (2)	0 (0)	410 (49)
	18–64 years	776 (4)	1556 (9)	6259 (35)	701 (4)	6 (0)	8731 (48)
	65–85 years	548 (6)	806 (8)	4349 (45)	375 (4)	0 (0)	3557 (37)
	More than 85	161 (7)	137 (6)	119 (54)	58(3)	3(0)	660 (30)
SEX	Female	698 (3.5)	1483 (7.5)	1277 (8.1)	691 (3.5)	14 (0.1)	9806 (49.5)
	Male	938 (6)	556 (3.5)	6242 (39.8)	556 (3.5)	16 (0.1)	6666 (42.5)

## Data Availability

The data used were extracted from a public database (https://www.adrreports.eu/, accessed on 1 May 2020).
